# Sucrose- and H^+^-Dependent Charge Movements Associated with the Gating of Sucrose Transporter ZmSUT1

**DOI:** 10.1371/journal.pone.0012605

**Published:** 2010-09-07

**Authors:** Armando Carpaneto, Hermann Koepsell, Ernst Bamberg, Rainer Hedrich, Dietmar Geiger

**Affiliations:** 1 Istituto di Biofisica-CNR, Genova, Italy; 2 Institut für Anatomie und Zellbiologie, Universität Würzburg, Würzburg, Germany; 3 Max-Plank-Institute for Biophysics, Frankfurt am Main, Germany; 4 Julius-von-Sachs-Institut, Molekulare Pflanzenphysiologie und Biophysik, Universität Würzburg, Würzburg, Germany; University of Heidelberg, Germany

## Abstract

**Background:**

In contrast to man the majority of higher plants use sucrose as mobile carbohydrate. Accordingly proton-driven sucrose transporters are crucial for cell-to-cell and long-distance distribution within the plant body. Generally very negative plant membrane potentials and the ability to accumulate sucrose quantities of more than 1 M document that plants must have evolved transporters with unique structural and functional features.

**Methodology/Principal Findings:**

To unravel the functional properties of one specific high capacity plasma membrane sucrose transporter in detail, we expressed the sucrose/H^+^ co-transporter from maize ZmSUT1 in *Xenopus* oocytes. Application of sucrose in an acidic pH environment elicited inward proton currents. Interestingly the sucrose-dependent H^+^ transport was associated with a decrease in membrane capacitance (C_m_). In addition to sucrose C_m_ was modulated by the membrane potential and external protons. In order to explore the molecular mechanism underlying these C_m_ changes, presteady-state currents (I_pre_) of ZmSUT1 transport were analyzed. Decay of I_pre_ could be best fitted by double exponentials. When plotted against the voltage the charge Q, associated to I_pre_, was dependent on sucrose and protons. The mathematical derivative of the charge Q versus voltage was well in line with the observed C_m_ changes. Based on these parameters a turnover rate of 500 molecules sucrose/s was calculated. In contrast to gating currents of voltage dependent-potassium channels the analysis of ZmSUT1-derived presteady-state currents in the absence of sucrose (I = Q/τ) was sufficient to predict ZmSUT1 transport-associated currents.

**Conclusions:**

Taken together our results indicate that in the absence of sucrose, ‘trapped’ protons move back and forth between an outer and an inner site within the transmembrane domains of ZmSUT1. This movement of protons in the electric field of the membrane gives rise to the presteady-state currents and in turn to C_m_ changes. Upon application of external sucrose, protons can pass the membrane turning presteady-state into transport currents.

## Introduction

For long distance transport from the side of production (source) in leaves to the user (sink) tissues, sucrose is loaded into the tube-like phloem network [1]. Phloem loading of sucrose, synthesized in photosynthetic cells (mesophyll) within the leaves, takes place in the sieve tube adjacent to companion cells. These transport-active cells appear to be interconnected via plasmodesmata to the sieve tubes. The flux and direction of sucrose is regulated by SUC/SUT type sucrose cotransporters [2], . Plant and animal sugar carriers shuttle their substrates in cotransport with protons or sodium ions, respectively. In contrast to animal cells, plants cells establish a pH gradient (acidic extracellular space) and very negative membrane potentials via plasma membrane proton pumps. From this electromotive force sucrose transporters gain energy to drive sucrose accumulation of more than 1 M. Recently detailed biophysical studies of ZmSUT1 revealed that this carrier is working like a perfect thermodynamic machine by which the proton gradient drives sucrose transport and vice versa on the basis of a 1∶1 H^+^:sucrose stoichiometry [7]. As a matter of fact ZmSUT1 is capable to mediate sucrose loading and unloading of the phloem [6] under physiological conditions. The ZmSUT1 behavior is in contrast to the animal counterpart SGLT1, which mediates sugar uptake only. These fundamental physiological differences between plant phloem- and animal blood stream sugar transporters are harbored in their unique structure-function relationships.

The knowledge about the transport cycle of plant sucrose transporters is, however, still very limited and dates back to the 1990s [8], [9]. Cotransporters characteristically display three main kinds of electrical activity. Besides the membrane current associated with the ion-coupled translocation of the organic substrate (transport-associated current, I_tr_), most cotransporters exhibit two further kinds of current in the absence of organic substrate: an ‘uncoupled’ (steady) current and a presteady-state current (I_pre_) [10], [11], [12], [13]. While the presteady-state current is best observed in the absence of substrate, it disappears when the substrate is present in saturating amounts [14], [15]. Using presteady-state measurements and voltage clamp fluorometry the Wright lab [11], [16], [17], [18] examined the transport cycle of the Na^+^ driven glucose cotransporter SGLT1 during sugar transport. They recorded changes in charge movement in response to rapid membrane potential jumps in the presence and absence of sugar. In Na^+^ buffers and in the absence of glucose, stepwise jumps in the membrane voltage elicited presteady-state currents (charge movements). Application of glucose, however, induced transport-associated inward Na^+^ currents and reduced the maximal charge movements (Q_max_). Presteady-state currents were completely inhibited by saturating sugar concentrations. Based on their results the authors developed an ordered eight-state model for the transport mechanism of SGLT1. Therein charge movements of SGLT1, giving rise to the observed presteady-state currents, were shown to be associated with the binding of sodium to the empty transporters (e.g. [16], [18]).

In addition to the conductance, the capacitance of biological membranes (C_m_) represents a basic electrical property. Changes in C_m_ arise from changes of the membrane surface area (endo- or exocytosis) or charge displacement in the electrical field of the membrane. The latter occurs e.g. when charged domains of integral membrane proteins undergo conformational changes or when ions bind to the protein within the electrical field of the membrane [19], [20], [21], [22], [23], [24], [25]. Thus charge displacements can turn membranes into highly nonlinear, time-varying capacitors. In this respect membrane proteins can be seen as insulators with specific dielectric properties and not only as conductors for ions/substrates.

In this study we combined C_m_ and presteady-state current measurements to elucidate the transport mechanism of ZmSUT1 in *Xenopus* oocytes. C_m_ measurements at various sucrose concentrations revealed a dose-dependent, reversible decrease of C_m_. Furthermore C_m_ was modulated by the membrane potential and the external proton concentration. To explore the molecular mechanism underlying the C_m_ changes, presteady-state currents (I_pre_) of ZmSUT1 transport were measured. Thereby we observed pronounced charge movements in the absence of the substrate. The decay rate constant (τ) of I_pre_ and the quantity of displaced charges (Q) depended on the voltage and proton concentration. The quantitative comparison of transport-associated currents and I_pre_ revealed that sucrose-induced transport currents can be predicted solely by the analysis of I_pre_ in the absence of sucrose. This relation suggests that charge movements at zero sucrose and sucrose-induced transport of protons arise from the same molecular mechanism.

## Results

### Ligands Induce Voltage Dependent Capacitance Changes in ZmSUT1

**Figure 1:**
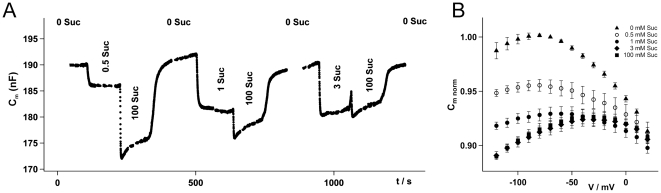
Sucrose dependent membrane capacitance changes in ZmSUT1 expressing oocytes. A) While continuously recording the membrane capacitance (C_m_) at −20 mV and pH 4, various external solutions (indicated in the figure) were applied. Upon perfusion with sucrose containing external media the C_m_ decreased in a dose dependent manner. B) When plotting C_m_ against the membrane potential it became apparent that at all tested voltages, C_m_ decreased in the presence of sucrose in a dose dependent manner (standard solution pH 4, sucrose concentrations are indicated in the figure, n≥4, ±SD).

**Figure 2:**
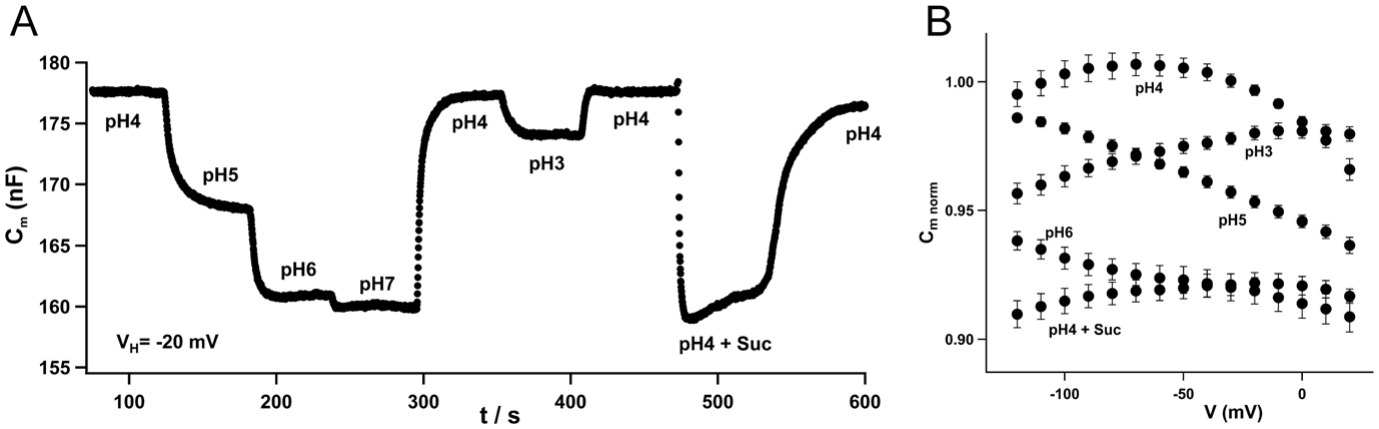
Capacitance changes in response to varying proton concentrations. A) Continuous recording of C_m_ at −20 mV and indicated pH values. When exchanging the bath solution by stepwise decreasing proton concentrations from pH 4 to 7 in the absence of sucrose, C_m_ decreased reversibly. Increasing the proton concentration from pH 4 to 3 at −20 mV led to a decrease of C_m_ as well. Subsequent application of saturating sucrose concentrations decreased C_m_ to similar levels as in the presence of neutral buffers. B) C_m_/voltage plot at various pH values without sucrose and at pH 4 in the presence of sucrose. Decreasing the proton concentration resulted in a shift of the C_m_ peak towards more negative membrane potentials. At pH 4 and in the presence of saturating sucrose concentrations C_m_ was depressed at all voltages tested (n≥5, ±SD). C_m_ values were normalized to values at pH 4 in the absence of sucrose.

The expression of ZmSUT1 in *Xenopus* oocytes and the application of 0.5, 1, 3 and 100 mM sucrose to the external medium induced dose-dependent inward currents (see Fig. 1 [7]). In addition to the current recorded via the two-electrode voltage-clamp technique (TECV) membrane capacitance changes were monitored [26]. In the absence of external sucrose at V = −20 mV, C_m_ for the depicted, representative oocyte was 190 nF. Upon addition of sucrose the membrane capacitance decreased reversibly in a dose-dependent manner (Fig. 1A). Upon application of external sucrose the membrane capacitance decreased at all voltages tested. In water injected oocytes C_m_ was not influenced by the addition of sucrose (data not shown). In absence of sucrose, voltage-dependent C_m_ changes were characterized by a bell-shaped curve with a maximum around –80 mV (Fig. 1B). During a stepwise increase in sucrose concentrations, the C_m_ drop saturated above 3 mM of the disaccharide at pH 4. ZmSUT1 represents a proton/sucrose cotransporter that drives sucrose at the expense of the proton gradient and *vice versa* [7]. We, thus, investigated whether and how external protons modulate C_m_. With the representative oocyte clamped to −20 mV and sucrose-free bath solution buffered to pH 4, C_m_ was 178 nF (Fig. 2A). A decrease in the proton concentration from pH 4 to pH 5, 6, and 7 was followed by a drop in capacitance characterized by a maximum at pH 4. The significant decrease of C_m_ upon a shift from pH 4 to pH 3, equivalent to 1 mM protons, was less pronounced than a similar shift of one pH unit from 4 to 5. The voltage-dependence of the membrane capacitance at different pH values appeared nonlinear (Fig. 2B). For a wide voltage range, from –120 to 0 mV, C_m_ peaked at pH 4 dropping at both lower (pH 5 and 6) and higher (pH 3) external proton concentrations. Moreover at pH 5 when compared to pH 3 an opposite voltage-dependence was recorded. Upon depolarization C_m_ decreased at pH 5 while it increased at pH 3. The application of saturating sucrose concentration lowered C_m_ at all voltages. In order to interpret the results obtained so far, we assumed that changes in membrane capacitance in ZmSUT1 expressing oocytes were associated with charge movements inside the transporter.

### Charge Movements in ZmSUT1 Generate Presteady-State Currents

**Figure 3:**
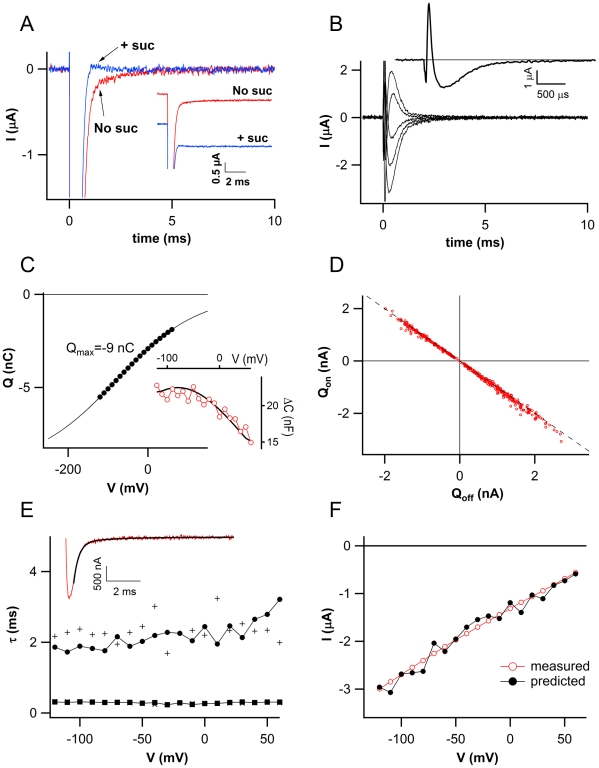
Presteady-state currents of ZmSUT1. A) Inset: currents recorded in the absence (No suc) and in the presence of saturating sucrose concentration (+suc). Same currents presented in the inset after forcing their stationary level to zero. Holding and pulse voltages were –20 and –80 mV respectively. B) Presteady-state currents versus time obtained after subtracting the stationary currents in the absence and in the presence of saturating sucrose. Holding voltage –20 mV, voltage pulses from +40 mV to –120 mV (step –40 mV). Inset: presteady-state current elicited by a voltage of –80 mV. C) Charge associated to presteady-state currents plotted versus the voltage. The charge was obtained from integration of the presteady-state currents (see Materials and Methods). The continuous line is the fit of Q with the Boltzmann function shown in Material and Methods with, in this oocyte, Q_max_ = −9 nC, V_h_ = −80 mV and s = 103 mV. Inset of panel C: Capacitance (empty symbols) measured using the method of Adrian and Almers (1976). The continuous line is the capacitance derived from the Q-V plot of panel C (see materials and methods). D) Q_on_ and Q_off_, the charge movements during the on and off response of the voltage steps obtained from the integrals of the presteady-state currents in 20 different oocytes, are plotted for various membrane voltages from –120 and +60 mV in 10 mV steps. The dotted line has a slope of 1. The linear fits (not shown) of the experimental points for each single oocyte give regression coefficients with a minimum value of −0.993, indicating very strong correlation. E) Time constants of the decay of the on and off presteady-state currents versus the applied voltage. The final decay of the presteady-state currents can be fitted by a two-exponential function with a fast and a slow τ (see Materials and Methods). The value of the fast time constants (▪ for the on and 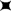 for the off presteady-state current) is under 1 ms and is limited by the speed of the voltage-clamp. Symbols • and + represent the slow time constants of the on and off presteady-state currents respectively. F) Transported (measured, empty symbol) and predicted (filled symbol) currents plotted against voltage for a single oocyte.

Charge movements (presteady-state currents) inside membrane proteins have been observed with ion channels [27]. These ‘presteady-state- or gating currents’ precede the opening and closing of voltage-dependent channels. To investigate the molecular basis of sugar-dependent modulation of C_m_ with ZmSUT1, we monitored voltage-dependent presteady-state currents of the sugar/proton co-transporter with high resolution. Thereby currents in response to membrane potential changes from a holding potential of −20 mV to −80 mV in the absence and presence of saturating sucrose concentration were compared (inset of Fig. 3A). As a matter of fact the stationary current in the presence of sucrose was significantly higher than in the absence of the sugar. These stationary sucrose-induced currents were named transport-associated currents (I_tr_). The transient current right after the voltage jump in the absence of sucrose, appeared slower in reaching the stationary level than with sucrose. This is better visible when subtracting the stationary currents (Fig. 3A). Presteady-state currents (I_pre_) associated to the ZmSUT1 activities were gained by subtracting the current transients at different voltages in the presence and absence of sucrose (Fig. 3B). The integral of the presteady-state currents over time plotted against the voltage reflect the associated charge-voltage curve (Fig. 3C). Note, that there is no significant difference from the “on” (integral of I_pre_ during the application of the voltage pulse) and the “off” charge (integral of I_pre_ during repolarisation; Fig. 3D).

When fitting Q vs V with the Boltzmann equation (continuous line in Fig. 3C, see materials and methods) a half activation voltage of –59±4 mV (mean±SEM, n = 20) was calculated. Using the derivative of the charge over time the capacitance change associated with the presteady-state current, was obtained (Fig. 3C inset, open circles). Note, that the calculated capacitance changes were well in agreement with the experimentally determined capacitance (black line) using small voltage steps according to the methods developed by Adrian and Almers [28] (see materials and methods, Fig. 3D). The good agreement between these two quantities guarantees that the mentioned experimental approach does not harbor significant problems of voltage-clamp due to the large potential pulses (ΔV up to 100 mV). The slope of the Boltzmann fit of 98±3 mV (mean±SEM, n = 20) is equal to KT/ezδ where K and T have the usual meaning, e is the elementary charge, z the valence of the moving charge and δ the fraction of the membrane field through which the charge moves; therefore zδ is equal to 0.26. If the moving charge is monovalent, then it moves 26% of the electrical distance from the external solution to the cytoplasmic side of the membrane. The decay of the presteady-state currents could be described by two exponential functions (inset of Fig. 3E). The exponential fitting identified two reliable time constants (Fig. 3E, see Materials and Methods). The fast component of less than 1 ms is biased by the speed of the clamp of the amplifier (squares). The slow time constant was around 2 ms at negative voltages and up to 3 ms at positive voltages (circles). The cross symbols in Fig. 3E represent the slow time constants derived from the exponential fitting of I_pre_ “off”. Their averages served as an estimate of the time constant of the “on” presteady-state current at the holding voltage of −20 mV.

### Relationship between I_pre_ and I_tr_

The transport-associated currents in the presence of external sucrose were apparently different to the presteady-state currents obtained in the absence of sucrose. The gating currents of voltage dependent ion channels appear independent from the presence or absence of the permeating ion [29]. Therefore these gating charges and the transport-associated currents are clearly distinct and not predictable. To test whether there exists a relation between I_pre_ in the absence of sucrose and I_tr_ in the presence of sucrose with ZmSUT1, we used the relationship I = Q/τ to predict the transport-associated currents solely from the I_pre_ parameters obtained without sucrose (cf. [30], ). Indeed we could obtain a very good agreement between the measured transport-associated currents (open circles) and predicted currents (filled symbols, Fig. 3F) indicating that there is an inter-relation between I_pre_ and I_tr_.

### ZmSUT1 Turns Over 500 Sucrose Molecules per Second

**Figure 4:**
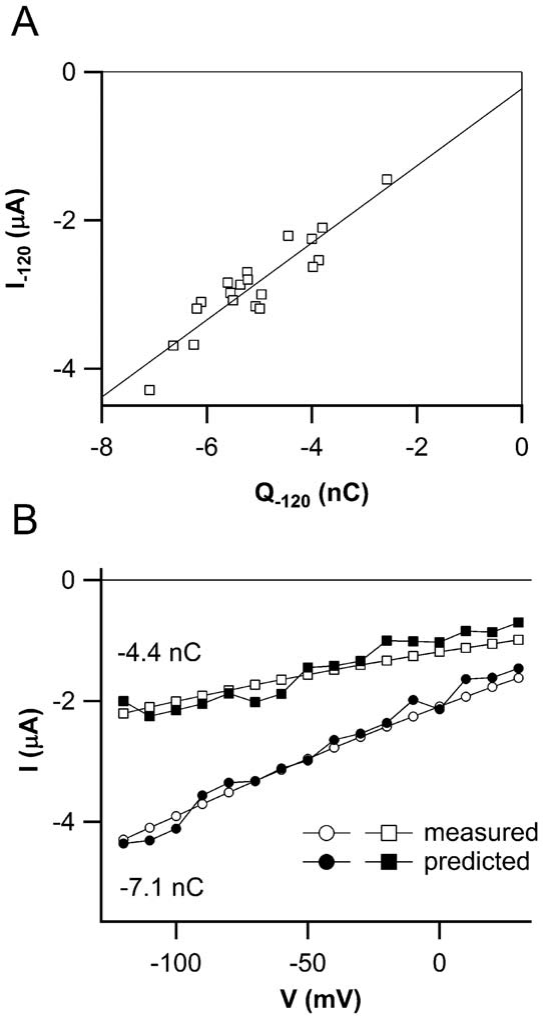
Properties of charge movement. A) Relation between I_-120_ and Q_-120_ for 20 individual oocytes; the linear regression (r = 0.92) gives a slope of 520±53 nA nC^−1^ or s^−1^. C) Transported (measured, empty symbols) and predicted (filled symbols) currents plotted against voltage for two oocytes with different level of ZmSUT1 expression.

In Fig. 4A we plotted the transport-associated currents (I_tr_), recorded at −120 mV in 20 oocytes differing in their individual level of ZmSUT1 expression, against the corresponding charges (Q_-120_). The linear fit showed a clear correlation between the two quantities (regression coefficient of 0.92), indicating that both I_tr_ and Q depend on the total number of transporters present on the membrane of the oocyte. Interestingly, and as expected from our previous analysis (Fig. 3F), the slope of the fit was 520 s^−1^ very close to the reciprocal of the slow time constant shown in Fig. 3E (about 500 s^−1^). The transport-associated currents of ZmSUT1-expressing oocytes with different levels of expression in Fig 4B were obtained by the subtraction of the currents in the absence from the currents in the presence of saturating sucrose concentration (open symbols). The filled symbols were again calculated using the ratio Q/τ based only on parameters obtained from the presteady-state currents in the absence of sucrose. The good agreement between the measured and predicted currents confirms that the prediction of I_tr_ from I_pre_ is independent from the expression level of ZmSUT1. In this respect, the turnover rate of the transporter is about 500 s^−1^ at negative (physiological) voltages which was calculated from the reciprocal of the slow time constant (see Fig. 3E) or from the slope of the linear fitting of I_-120mV_ vs Q_-120mV_ (Fig. 4B). Moreover we can estimate the average number of transporters from the equation: Q_max_ = Nezδ where Q_max_ is the total charge movement, N the number of transporter per oocyte, e the elementary charge and zδ was obtained by the Boltzmann analysis performed above. Considering a mean Q_max_ of 8 nC, N is 2×10^11^ transporter molecules per oocytes, a value well in line with other transporters (i.e. GAT1 [15], KAAT1 [14]) confirming the capability of the oocyte system to express foreign proteins at very high densities. In our experiments the average capacitance of oocytes was 170 nF. Using the specific capacitance of 1 µF/cm^2^ of biological membranes we obtain an average oocyte area of 1.7×10^7^ µm^2^ and a transporter density of about 10^4^/µm^2^. If each transporter effectively covers an approximate area of 5 nm^2^ (Abramson, 2003), ZmSUT1 would cover about 25% of the total oocyte area.

### Sucrose Affects ZmSUT1-Derived Presteady-State Currents

**Figure 5:**
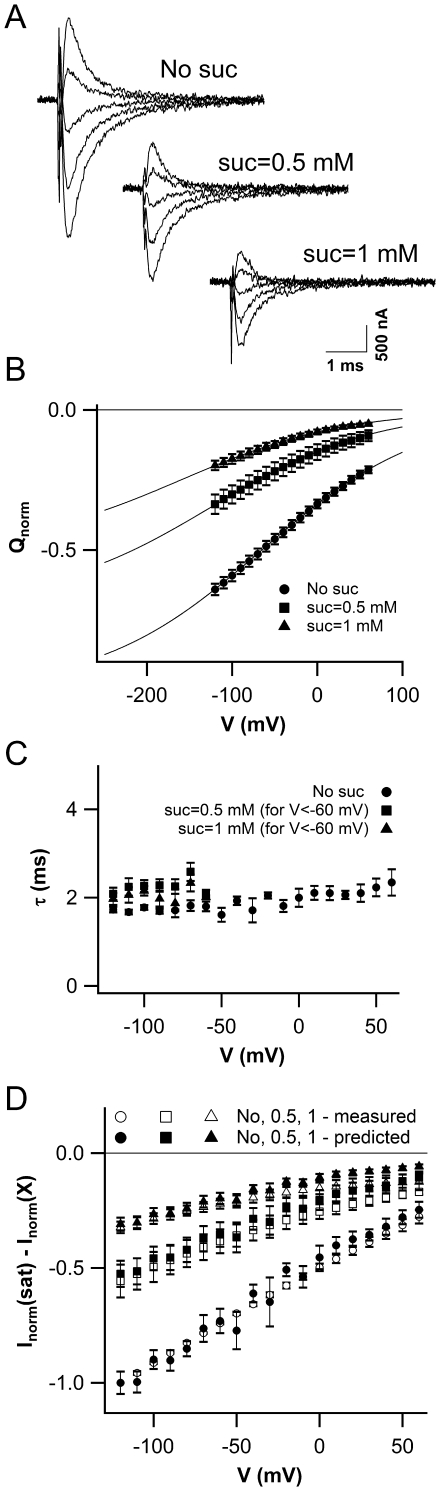
Sucrose dependence of ZmSUT1 presteady-state currents. A) Presteady-state currents of a single oocyte at varying external sucrose concentrations. For the sake of clarity only currents recorded from +40 to –120 mV, in 40 mV step, are shown; the holding voltage was –20 mV. B) Charge associated to presteady-state currents versus voltage at different sucrose concentrations. The continuous lines are fit of Q using a Boltzmann function (see Materials and Methods). Data for each oocyte were normalized to the maximum charge obtained in the absence of external sucrose, Q_max_(No suc). The fits gave the following parameters (mean±SEM, n = 4): no external sucrose: V_1/2_ = −65±8 mV, s = 96±5 mV; 0.5 mM sucrose: V_1/2_ = −123±17 mV, Q_max_(suc = 0.5 mM)/Q_max_(No suc) = 0.69±0.02; 1 mM sucrose V_1/2_ = −159±20 mV, Q_max_(suc = 1 mM)/Q_max_(No suc) = 0.50±0.02. At 0.5 and 1 mM sucrose the slope s of the fit was fixed at the value obtained at 0 external sucrose. C) I_sat_ – I_X_ against the applied membrane potential. I_sat_ is the current at saturating external sucrose concentration; I_x_ are the currents in the presence of 0, 0.5 mM and 1 mM external sucrose. Empty and filled symbols refer to the measured and predicted currents respectively. The following relationship was used in order to evaluate the predicted currents: I_sat_ – I_X_ = Q_X_/τ where X represents the external sucrose concentration and τ is the slow time constant at zero external sucrose.

The presteady-state currents of ZmSUT1, obtained in a similar manner as those shown in Fig. 3, appeared modulated by the external sucrose concentration. Application of 0.5 and 1 mM external sucrose resulted in a sucrose-dependent reduction of I_pre_ (Fig. 5A). The charge associated to I_pre_ versus voltage decreased upon increasing the external sucrose concentration (Fig. 5B) and its derivative was in agreement with the measured capacitance (Fig. S1A). There was, however, no significant effect of external sucrose at concentration equal to 0.5 and 1 mM on the slow time constant (Fig. 5C). The relationship I = Q/τ previously used to predict the transport currents solely from the knowledge of the presteady-state currents can be written as: 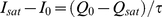
(1)where I_sat_ and I_0_ represent the transport-associated currents in the presence and absence of saturating sucrose, Q_nosuc_ and Q_sat_ are the charges associated to the transient component of the transporter current in the absence and presence of the sugar and τ represents the slow time constant of the exponential fitting of I_pre_. Equation 1 can be extended to predict the ZmSUT1 response to given external sucrose concentration (X):
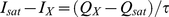
(2)where I_X_ and Q_X_ are respectively the stationary current and the charge associated to the transient component of the ZmSUT1 current in the presence of a sucrose concentration equal to X. To compare the measured and the predicted transport currents the I_sat_ – I_X_ values, obtained from the transport-associated currents, were normalized (see Materials and Methods) and plotted as a function of voltage (open symbols, Fig. 5C). The filled symbols were calculated using equation 2 with parameters obtained from the experimentally recorded I_pre_ (for the normalization procedure see Materials and Methods). Please note the good agreement between the experimental and predicted data over a broad voltage range. This agreement strongly suggests a similar origin of the presteady-state currents and the transport-associated currents. One might thus conclude that I_pre_ in the absence of sucrose reflect proton movements from an external to an inner position of the transporter and that I_tr_ reflects the proton permeation across the membrane when sucrose is added [7].

### Protons Affect the Gating of ZmSUT1

**Figure 6:**
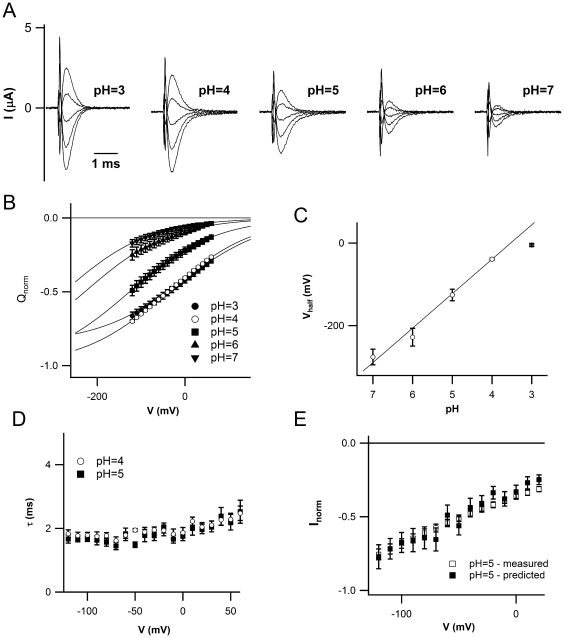
pH dependence of ZmSUT1 presteady-state currents. A) Presteady-state currents of a single oocyte at varying external pH. Holding voltage –20 mV, voltage pulses from +40 mV to –120 mV (step –40 mV). B) Normalized charge associated to presteady-state currents at different external pH versus voltage. For the details of the pH analysis see Materials and Methods. Q_max_(pH = 3)/Q_max_(pH = 4) = 0.85±0.03. Data were obtained by four different oocytes. C) Half activation voltages (V_1/2_) obtained by data in panel B versus external pH. The slope of the linear fit from pH 7 to 4 is equal to 83±5 mV. D) Slow time constant τ recorded at pH 4 and 5 plotted against applied voltage. E) Predicted (filled symbols) and measured (empty symbols) transported currents versus voltage (see Materials and Methods for details).

When the proton concentration was reduced from pH 4 to pH 7 a strong reduction of I_pre_ was observed (Fig. 6A). To gain the voltage dependence of this process we plotted the I_pre_-associated charge Q in different external pH buffers against the applied voltage (Fig. 6B). Plotting the half activation voltage, obtained in panel B, against decreasing external proton concentration (Fig. 6C) visualized the shift of Q(V) towards negative voltages. A linear fit from pH 7 to pH 4 resulted in a slope of 83±5 mV per pH unit (n = 4). These results are in agreement with our notion that protons generate ZmSUT1 presteady-state currents. As expected, the derivative of the charge is in line with the actually measured capacitance as shown in Fig. S1. The slow time constants of I_pre_ at pH 5 and 4 are not significantly different (Fig. 5D). In line with our experiments at pH 4, the agreement between the predicted and measured stationary currents at pH 5 were satisfactory (Fig. 6, see Materials and Methods for details).

## Discussion

The capacitance (C_m_) of biological membranes represents a basic electrical property. Using the paired ramp technique [26] we could in real-time monitor changes in C_m_ of *Xenopus* oocytes over a broad range of physiological conditions such as pH-, sucrose- and membrane potentials changes. The robustness of the C_m_ measurement technique allowed us to measure C_m_ even under conditions where presteady-state measurements were biased by the speed of the voltage-clamp and therefore beyond the resolution limit of the method.

The Wright lab in 1996 recorded presteady-state currents of the potato H^+^/sucrose cotrasporter StSUT1 [8]. In contrast to ZmSUT1 those of StSUT1 appeared sucrose independent. In the presence of saturating extracellular sucrose steady state currents increased without affecting the transient component. Compared to water-injected oocytes, StSUT1 expressing ones exhibited a large, pH-dependent transient component. Acidification of the extracellular solution did not change both the total charge (Q_max_) and the apparent valence (z) but shifted the Boltzmann curve 30 mV more negative. Therefore it was not possible with the weak StSUT1 activity in oocytes to correlate the effect of the membrane voltage and the H^+^ concentration on presteady-state kinetics with those of transport currents (steady-state). How can this discrepancy to the situation with ZmSUT1 be explained? The transient components in StSUT1 could reflect structural arrangements of the carrier protein. With ZmSUT1 expressing oocytes, sucrose-independent presteady-state currents were not apparent. The transient component in the presence of saturating sucrose was rather comparable to those of water injected oocytes. Stationary currents with StSUT1 injected oocytes were 20–50 times smaller than those obtained with ZmSUT1. The discrepancy between *Zea maize* and *Solanum tuberosum* transporter may thus reflect a problem of resolution only.

Taking advantage of the high transport activity of ZmSUT1, we could measure presteady-state currents with a shift of the associated charge by about 80 mV upon acidification. The I_pre_ voltage-dependence of ZmSUT1 is in agreement with the range of physiological membrane potential of the plant cell. E.g. at an extracellular pH of 5–5.5, the half-activation voltage V_1/2_ of I_pre_ is more negative than –120 mV. Since ZmSUT1 is capable to mediate sucrose loading as well as unloading [7], the slope of about 100 mV indicates that the H^+^/sucrose cotransporter might indeed operate in a wide voltage range.

The experimental analysis of the sugar and proton dependent ionic currents associated with the gating and solute movement of ZmSUT1 point to the following transport mechanism: the proton entrance into the transporter is far from the electric field (indicated by the voltage-independence of τ) and was the rate limiting step with respect to the overall transport mechanism under our specific experimental conditions. It is worth noting that this rate limiting step is not necessarily caused by the diffusion and/or binding of the protons inside ZmSUT1 but could be influenced by the accessibility of the proton binding site due to conformational changes of the transporter. As proposed by Kaback's lab (e.g. [32], [33]) for ion-coupled cotransporters, in the absence of substrate the transporter can have two main conformations for the proton binding site, respectively facing the extracellular (outward-facing) and the cytosolic side (inward-facing). The slow time constant measured in this work could represent the transition between these two states. In the absence of sugar the proton is trapped inside the transporter and can move up to 25% of the electrical distance across the membrane. This voltage-dependent movement is the origin of the presteady-state currents. In this case the transporter can be seen as a pure capacitor and the protons are the relative charges. When saturating sucrose concentrations are present at the extracellular surface of ZmSUT1, the proton inside the transporter is released into the cytoplasm from the inward-facing conformation. When crossing the barrier one proton moves one sugar molecule into the cytoplasm [7]. Very likely this step is associated with a conformational change of the transporter [11], [32]. From our data we predict that the conformational change that moves the protons from the extracellular to the cytosolic side in the presence of sucrose has to be faster than 2 ms. In this scenario the transporter can be seen as a pure resistor and the protons are the charges giving rise to the electrical transport current. Under conditions of limiting sugar in the extracellular milieu the transporter behaves like a capacitor as well as a resistor. Furthermore the relationship between presteady-state and transport-associated currents suggests that the proton entry into the electric field across ZmSUT1 is not influenced by the presence of external sucrose. This link points to a common origin of the presteady-state and the transport-associated currents, which is underpinned by the fact that the reduction of the external H^+^ concentration decreased both the presteady-state and the transport-associated currents.

Transporters both from animal (SGLT1, PepT1, [24], [34]) and plant kingdoms (STP1, [35]) are characterized by presteady-state currents which are apparent in the absence of external substrate and disappear under substrate saturation. In GAT1 and GlyT1 transporters (respectively a sodium/chloride GABA and glycine cotransporter present in synapses of animal cells) a possible inter-conversion between the two kinds of currents was recently explored [30], [31], [36]. Presteady-state currents of GAT1 and GlyT1 result from the displacement of Na^+^ or chloride ions within the electrical field of the membrane traveling between the extracellular space and a binding site in the protein. Interestingly Fesce et al. (2002, [30]) and Cherubino et al. (2010, [36]) could predict transport-associated currents in the presence of GABA or glycine by analyzing presteady-state currents in the absence of substrate. For these animal cotransporters it was proposed that in the absence of substrate sodium/chloride could move, with a rate-limiting speed represented by τ, from an external to an internal site of the transporter generating the presteady-state currents. Upon addition of substrate the ‘trapped’ ion is able to cross the membrane with the same rate-limiting step. Our analyses with ZmSUT1 point to a similar relationship between the transient current component and the transport-associated currents. Future studies will thus need to focus on the question whether similar relationships between charge movement and transport-associated currents found in different cotransporter types have common grounds.

## Materials and Methods

An ethics statement is not required for this work.

### TEVC analysis in *Xenopus* Oocytes

ZmSUT1 cRNA was prepared using the mMESSAGE mMACHINE™ RNA Transcription kit (Ambion Inc., Texas, USA). Oocyte preparation and cRNA injection have been described elsewhere [37]. In two-electrode voltage-clamp studies oocytes were perfused with a standard-solution containing 30 mM KCl, 1 mM CaCl_2_, and 1.5 mM MgCl_2_ based on Tris/Mes buffers for pH values from 5.5 to 7.5 or based on citrate/Tris buffers for the pH values 3.5 to 5.0. Solutions were adjusted to 220 mosmol kg^−1^ using D-sorbitol.

### Transport Current and Membrane Capacitance Measurements

Simultaneous measurements of transport currents (I_tr_) and membrane capacitance (C_m_) were performed using an TEC-10X amplifier (NPI Electronic, Tamm, Germany) controlled by the PULSE and X-Chart software (HEKA Electronics). C_m_ was measured using a previously described paired ramps approach [26]. Sucrose-induced proton currents and sucrose-induced capacitance changes were recorded at −20 mV while the oocytes were super-fused with different external solutions. When the bath medium was completely exchanged C_m_ and I_tr_ were determined in the voltage range between +20 and −120 mV in 10 mV decrements.

Alternatively we use the method developed by Adrian and Almers (see also [14]): a small voltage step of 20 mV (ΔV) and duration of 20 ms was superimposed to a given applied voltage. The amount of moved charge ΔQ was obtained by integrating the current transient. The slope of the membrane capacitance at the applied voltage was approximated by the ratio ΔQ/ΔV. We verified that the two methods of measuring membrane capacitance described above gave similar results (data not shown).

### Presteady-State Currents

Presteady-state currents were obtained using the two-electrode voltage-clamp technique (TECV) subtracting the traces at saturating sucrose concentration (typically 100 mM) and forcing the steady level at the end of each voltage pulse to zero. The holding potential was maintained at –20 mV and voltage pulses of 20 ms from 60 mV to –120 mV in –10 mV steps were applied. Signals were sampled at 100 kHz and filtered at 20 kHz.

The charge (Q) associated to each presteady-state currents was obtained using the following procedure: a) the charge Q_exp_ was calculated by the integral of the presteady-state currents and fitted by the Boltzmann equation Q_max_/(1+ exp[(V-V_1/2_)/s]) + Q_i_, where Q_i_ represents the charge offset, Q_max_ the maximum charge, V_1/2_ the half-activation voltage and s is linked to the slope of the Boltzmann curve at V = V_1/2_. The charge Q was Q_exp_ – Q_i_. The “on” charge, the charge calculated when the voltage pulse was applied, and the “off” charge, calculated when the potential was again set to the holding value, did not differ considerably (see Fig. 3D).

The last part of the decay of the presteady-state currents was fitted by the two exponential function I_fast_ exp(−t/τ_fast_) + I_slow_ exp(−t/τ_slow_). In order to verify the reliabilty of τ, the starting time of the fit was increased in step of 50 µs; in each step the fitting procedure was applied (up to 10 steps) and τ was calculated as the average value. When the relative error (SD/mean) of τ was greater that 10% the point was discarted; this was the case for sucrose concentrations equal to 0.5 and 1 mM at voltages more positive than –60 mV and external pH of 7, 6 and 3 (see Fig. 5 and 6).

To obtain τ at the holding voltage (V_hold_) a similar fitting procedure was performed on the off charge at voltages different from V_hold_. τ(V_hold_) was calculated as the average value of τ_off_(V). The transported currents induced by sucrose were calculated as the difference of the stationary currents in the presence and the absence of sucrose respectively. The predicted current was obtained using the following relationship: I = Q/τ. Changes in capacitance associated with the presteady-state currents (C_p_) were evaluated as the derivative of Q with respect to V and compared with the measured capacitance. Only the experiments with C_pred_∼C_m_ were considered.

#### Analysis of the sucrose dependency

For the analysis of the sucrose dependency shown in Fig. 5 presteady-state currents at 0, 0.5 and 1 mM sucrose were obtained from the experimental currents subtracting the traces at saturating sucrose concentration and forcing the steady level to zero at the end of each voltage pulse. The integral of the presteady-state currents from a single oocyte was fitted by the Boltzmann equation shown above. At 0.5 and 1 mM sucrose the slope s of the fit was fixed at the value obtained at 0 mM external sucrose. Q_X_ was calculated as (Q_exp_(X) - Q_i_(X))/Q_max_(0) with X equal to 0, 0.5 and 1 mM external sucrose. The predicted currents were calculated using equation 2 (see Results session) with the slow time constant obtained in the absence of external sucrose; as a matter of fact τ was not significantly affected by external sucrose concentrations of 0.5 and 1 mM (see Fig. 5C). The predicted and measured currents for a single oocyte were normalized at the value of (I_sat_- I_0_) evaluated at –120 mV. Data from four different oocytes were averaged.

#### Analysis of the proton dependency

The sequence of external solutions perfusing the ZmSUT1-expressing oocyte was the following: 1) from pH 3 to 7 in steps of one pH unit in absence of external sucrose, 2) going to pH 4, in the absence of external sucrose and finally 3) applying saturating concentration of external sucrose at pH 4. Presteady-state currents were obtained using the standard procedure after subtraction of traces recorded at pH 4 in saturating external sucrose. The charge (Q) was obtained by the integral of the presteady-state currents and fitted by the Boltzmann function shown above. The slope s was fixed to 98 mV, the average value at pH 4 from 20 different oocytes, see text. The maximum charge, Q_max_, was fixed at the values obtained at pH 4; at pH 3 Q_max_ was considered as a free parameter of the fit and was normalized to the value at pH 4. We used the relationship I = Q/τ, where Q and τ are the charge and the slow time constant at pH 4 and 5, to calculate I(pH = 5) i.e. the transported current at pH 5. I(pH = 5) was divided by I(pH = 4, V = −120 mV) and compared, as shown in Fig. 6E, with the stationary currents at pH 5 normalized to the value of the current at pH 4 and V = −120. Stationary currents were obtained by a different set of experiments.

Data are given as mean±SEM together with the number, n, of different oocyte investigated.

The TECV set-up was equipped by a home-made system to control the temperature. Experiments were performed at a temperature of 22.0±0.1°C.

## Supporting Information

Figure S1 The empty symbols are the capacitance measured using the method of Adrian and Almers (1976) in response to different external sucrose (A) or pH (B) conditions. Continuous lines were derived from the Q-V plot of Fig. 5B in (A) and of Fig. 6B in (B).
(4.27 MB TIF)
