# Supplementary figures and images for: Sucrose- and H+-Dependent Charge Movements Associated with the Gating of Sucrose Transporter ZmSUT1
Source: PLoS One. 2010 Sep 7;5(9):e12605. doi: 10.1371/journal.pone.0012605 (PMC2935479; doi:10.1371/journal.pone.0012605)

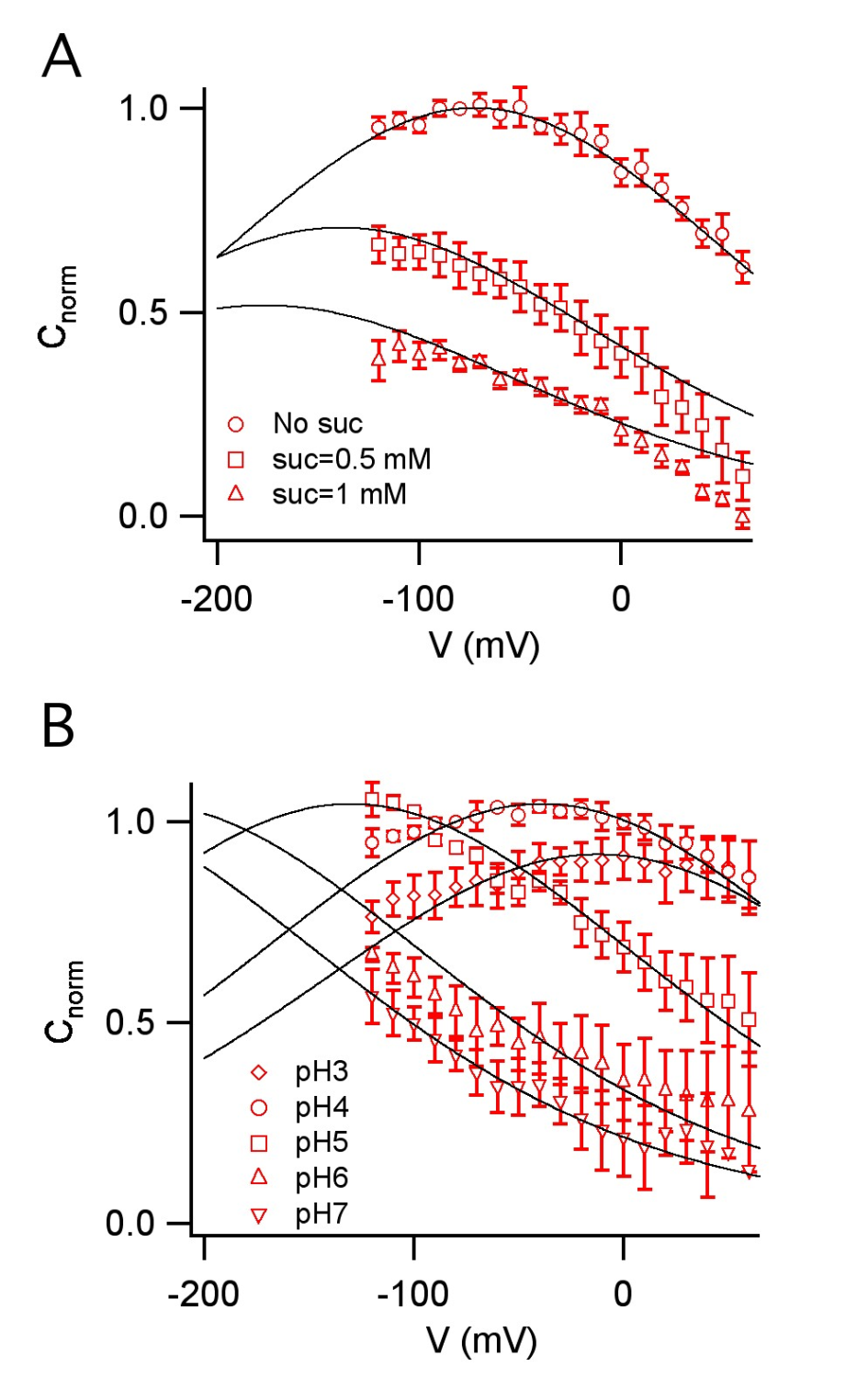

Supplement: Figure S1 — The empty symbols are the capacitance measured using the method of Adrian and Almers (1976) in response to different external sucrose (A) or pH (B) conditions. Continuous lines were derived from the Q-V plot of Fig. 5B in (A) and of Fig. 6B in (B). (4.27 MB TIF) [file pone.0012605.s001.tif]
